# Systems Biology Approaches to Understanding the Human Immune System

**DOI:** 10.3389/fimmu.2020.01683

**Published:** 2020-07-30

**Authors:** Bhavjinder K. Dhillon, Maren Smith, Arjun Baghela, Amy H. Y. Lee, Robert E. W. Hancock

**Affiliations:** ^1^Centre for Microbial Diseases and Immunity Research, University of British Columbia, Vancouver, BC, Canada; ^2^Molecular Biology & Biochemistry Department, Simon Fraser University, Burnaby, BC, Canada

**Keywords:** systems biology, multi-omic integration, transcriptomics, innate immunity, immune ontogeny, host-pathogen interaction, drug discovery and repurposing, systems vaccinology

## Abstract

Systems biology is an approach to interrogate complex biological systems through large-scale quantification of numerous biomolecules. The immune system involves >1,500 genes/proteins in many interconnected pathways and processes, and a systems-level approach is critical in broadening our understanding of the immune response to vaccination. Changes in molecular pathways can be detected using high-throughput omics datasets (e.g., transcriptomics, proteomics, and metabolomics) by using methods such as pathway enrichment, network analysis, machine learning, etc. Importantly, integration of multiple *omic* datasets is becoming key to revealing novel biological insights. In this perspective article, we highlight the use of protein-protein interaction (PPI) networks as a multi-omics integration approach to unravel information flow and mechanisms during complex biological events, with a focus on the immune system. This involves a combination of tools, including: *InnateDB*, a database of curated interactions between genes and protein products involved in the innate immunity; *NetworkAnalyst*, a visualization and analysis platform for InnateDB interactions; and *MetaBridge*, a tool to integrate metabolite data into PPI networks. The application of these systems techniques is demonstrated for a variety of biological questions, including: the developmental trajectory of neonates during the first week of life, mechanisms in host-pathogen interaction, disease prognosis, biomarker discovery, and drug discovery and repurposing. Overall, systems biology analyses of omics data have been applied to a variety of immunology-related questions, and here we demonstrate the numerous ways in which PPI network analysis can be a powerful tool in contributing to our understanding of the immune system and the study of vaccines.

## Introduction

In the field of immunology, a systems biology approach is necessary to understanding the immune response to vaccination, infection and diseases, since these involve complex interactions between a large number of genetic, epigenetic, physiological and environmental factors. Systems-level strategies can ultimately be applied to better understand the molecular changes in humans upon exposure to a vaccine or an immunotherapeutic, to understand the mechanisms underlying disease or pathogenesis, and to characterize the effect(s) of specific challenges to the immune system (1–5). Omics technologies offer the ability to measure such aspects in an unbiased way that is high-throughput and cost-effective. Several omics methods have been employed in the context of systems vaccinology (3), including but not limited to, whole genome sequencing (genomics), RNA-Seq for measuring mRNA levels (transcriptomics), high-throughput mass spectrometry for measuring protein levels (proteomics) and metabolite levels (metabolomics), CHiP-Seq for determining transcription factor binding sites, ATAC-Seq to identify DNA modification sites (epigenomics), 16S rRNA sequencing for microbiota profiling (microbiomics), and equivalent omics analyses performed at the single-cell level. Recently, there has also been a growing effort to obtain multiple omics profiles in the same individuals, since shared insights across omics datasets strengthens links between underlying biological mechanisms and responses of interest, and can provide more reliable interpretation of gene function, higher-level changes and novel insights not observed in single-omics studies (6–8). Overall, biological samples can be manipulated to generate numerous omics datasets, and can be applied to study how our immune systems elicit effective, therapeutic and/or pathological responses.

A key challenge in systems biology is building the appropriate bioinformatics tools to integrate omics datasets, ultimately enabling the correlation of global changes with the underlying biological events that drove those changes. Statistical and machine learning approaches have been applied to omics datasets [reviewed previously (9–11)] to identify sets of molecular features that (i) are dysregulated/correlated with observed phenotypes, (ii) can be used as biomarkers to predict observed phenotypes, or (iii) can be targeted by drugs for improved therapies. A wide array of tools are available to run single- or multi-omics analysis pipelines (12), including commercial platforms and more recently published “self-serve” platforms [e.g., OmicsNet (13), OmicsPlayground (14)]. Typically, such methods interrogate information in either a supervised or unsupervised manner; supervised methods identify differences between labeled omics data from different conditions (e.g., responders vs. non-responders or treated vs. untreated) while, unsupervised methods reveal global patterns of gene dysregulation without any labels.

Downstream characterization of dysregulated molecules can further our understanding of underlying biological mechanisms at play. This can be achieved by interrogating curated functional genomics information from databases of gene ontologies (functional descriptions), pathways, known interactors, transcription factor binding sites (TFBS) upstream of dysregulated genes, etc. through various enrichment analyses. However, a large proportion of genes have not been assigned to canonical pathways in pathways databases (such as KEGG or Reactome), so pathway enrichment limits the ability of such approaches to reveal novel insights (15).

The use of biological networks is a powerful approach to integrate multi-omics data to identify novel biological insights (15–18). To characterize the role of individual molecular features in larger cellular processes and global changes using networks involves either overlaying omics data on experimentally-derived known networks [e.g., protein-protein interaction (PPI) networks], or by inferring networks directly from the data [e.g., co-expressed genes (19)], the strengths/limitations of which have been reviewed previously (15). A few commonly used biological networks along with related resources and tools are summarized in Table 1. The application of PPI networks to interrelate dysregulated genes is a very powerful method for revealing the systems-level flow of information through key hubs (highly connected protein nodes) and subnetworks. Because PPIs include direct, metabolic, and regulatory interactions between proteins, they essentially chart potentially biologically relevant, i.e., functional, interconnections. This can enable the determination of emergent properties, which are essentially new biological insights into the processes driving the observed transcriptional differences. The results from a PPI network analysis are always framed as hypotheses rather than knowledge *per se*, and must be eventually tested using downstream wet lab experiments (15).

**Table 1:** Examples of functional biological information that can be represented using networks, along with corresponding databases/repositories and supplementary data analysis tools that can be used to assess the functional data in high-throughput omics datasets.

**Type of functional information**	**Select databases/ repositories**	**Supplementary Omic-data analysis tools**
Metabolic pathways, reactions and associated enzymes and transporters	Kyoto Encyclopedia of Genes and Genomes (KEGG) (20)	MetaBridge (21)
	Reactome (22)	OmicsPlayground (14)
	Panther pathway database (23)	ReactomePA (24)
	Gene Ontology (25)	SIGORA (26)
	Edinburg Human Metabolic Network (27)	iMAT (28)
	Recon3D (29)	INIT (30)
	iHsa (31)	mCADRE (32)
	BioModels (33)	TIMBR (31)
Gene regulatory networks (interactions between transcription factors and their target genes)	Encyclopedia of DNA elements (ENCODE) (34, 35) JASPAR (36) TRANSFAC (37)	MRNET (38) ARACNE (39) iRegulon (40) dynGENIE3 (41)
Protein-protein interaction (PPI) networks (includes direct, metabolic, and regulatory interactions)	International Molecular Exchange (IMEx) Consortium (42), which includes: InnateDB (43, 44) Biomolecular Interaction Network Database (BIND) (45) Database of Interacting Proteins (DIP) (46) Molecular INTeraction Database (MINT) (47) MIntAct (48) The Biological General Repository for Interaction Datasets (BioGRID) (49)	NetworkAnalyst (50–52) OmicsNet (13) PPIExp (53)
Signaling networks (interactions involved in a cell's response to its environment)	KEGG (20) Reactome (22) STKE (54) TRANSPATH (55)	ReactomePA (24)
Drug targets (interactions between drugs and their cellular targets)	DrugBank (56) Therapeutic target database (TTD) (57) SuperTarget (58) STITCH (59) ChEMBL (60) BindingDB (61) KEGG (20)	DINIES (62)

In this article, we provide an overview of the philosophies and methodologies that can be employed in the analysis of omics data, especially with regards to integration of omics datasets using an unsupervised network analysis approach. Examples are provided of how such analyses enable novel hypothesis generation for: (a) immune system development, (b) mechanisms of host-pathogen interactions, (c) discovery of mechanism-based biomarkers, and (d) strategies to define prospective new interventions based on drug repurposing. While the methods are somewhat biased toward the study of innate immune and inflammatory responses, it is worth mentioning that “innate immunity instructs adaptive immunity” (63) in that (i) the effectors of adaptive immunity are often innate immune mechanisms, (ii) many of the pathways involved are the same, and (iii) vaccine adjuvants that improve adaptive immune responses boost innate immunity. Therefore, the tools we describe have value in investigating adaptive immunity as well as human genetic diseases/conditions with an underlying inflammatory pathology.

## Systems Tools for Network-Based Analyses Using PPIs

InnateDB (43, 44) and other International Molecular Exchange (IMEx) consortium databases (42) provide the basis for understanding biological connections in cells according to known interactions between molecular elements, such as proteins. InnateDB is a publicly available database (www.innatedb.com) focused on elucidating the genes, proteins, and molecular “interactome” of the innate immune response, with an emphasis on curation of experimentally-validated PPIs and signaling pathways in human, mouse and bovine. The interactome can be used to understand the interplay between multi-omics datasets that measure different parts of a larger system of physical, metabolic, and regulatory networks. For example, human TRAF6 and MyD88 are usually defined as having a role in the major TLR4 to NFκB signaling pathway of innate immunity. However, in InnateDB, they are experimentally documented to interact with 398 and 129 proteins, respectively, in humans. This means that there is a massive potential for these proteins to bridge and/or participate in multiple biological pathways when activated by innate immune stimuli.

InnateDB is an important tool in immunology as evidenced by the >6,000,000 hits from more than 55,000 visitors annually. While all known pathways (>3,500) and molecular interactions (318,000 in human) are present, the emphasis on innate immunity is achieved through the contextual review, curation and annotation of molecular interactions and pathways involved in innate immunity. To date, the InnateDB curation team has reviewed more than 5,200 publications annotating >27,000 molecular interactions of >9,400 separate genes in rich detail including annotation of the cell, cell-line and tissue type; the molecules involved; the interaction detection method; etc. By including interaction and pathway data relevant to all biological processes, a much broader perspective of innate immunity can be achieved, especially since an effective innate immune response requires the coordinated efforts of many important processes including the endocrine, circulatory, and nervous systems (64). Additionally, it becomes possible to investigate any biological signaling process of interest beyond the immune system, as well as inflammation and adaptive immunity.

InnateDB facilitates systems-level analyses by enabling the integration, analysis and visualization of user-supplied quantitative data, such as gene expression data, in the context of molecular interaction networks and pathways. This includes the statistically robust analysis of overrepresented pathways, interactomes, ontologies, TFBS, and networks. One can, for example, refine the network to show only molecular interactions between a list of differentially expressed (DE) genes (and their encoded products) or view all potential interactors regardless of whether they are DE. This can aid in the identification of important nodes that may not be regulated transcriptionally or which are expressed at an earlier or later time. Networks derived from InnateDB can be interactively visualized using the Cerebral plug-in for Cytoscape (65) to generate biologically intuitive, pathway-like layouts of networks, or in a more recently developed tool, NetworkAnalyst (50–52). NetworkAnalyst is an extremely fast network analysis and visualization tool for the analysis of gene expression data in the context of PPI networks. In addition, MetaBridge (21) is a tool that can be used for the integration of metabolite-protein interactions into these existing networks. In combination, these tools can be used to perform multi-omics integration of transcriptomics, proteomics, and metabolomics data in an unsupervised manner.

In addition to these outlined methods, there are bioinformatics tools available for performing other types of network analyses specifically for studying the immune system. Examples include immuneExpresso (66), a data mining tool built as part of Immport to capture inter-cell interactions, and Ontogenet (67), a component of the ImmGen database enabling construction of gene regulatory networks based on sets of co-expressed genes. Such tools can be useful in revealing novel inter-cell interactions or regulatory factors, respectively, but ultimately may be too limited in scope for a systems-level analysis. Thus, we focus here on how PPI-based network analysis tools can be applied to better understand human health and disease.

## Mechanistic Insights Into Human Immune Development

Most recently, as a part of the EPIC-HIPC consortium, we published a study that revealed a robust developmental trajectory of immune ontogeny during the first week of life in newborns using a multi-omics integration approach (9). Transcriptomic, proteomic, and metabolomic data were derived from <1 ml of blood collected from West African (The Gambia) neonates at two time points: day of life (DOL) 0 and a second DOL, either 1, 3, or 7.

Importantly, through this study, we were able to show that multi-omics integration using PPI networks (through NetworkAnalyst, InnateDB, and MetaBridge) provided similar biological insights, but greater depth, when compared to data-driven supervised integration approaches [namely, DIABLO (68) and Multifactorial Response Network (MMRN) (69, 70)]. Major observations from this study revealed that the first week of life is highly dynamic; DOL0 and DOL1 were quite similar with few DE genes, but by DOL3, 1,125 DE genes were detected, and 1,864 DE genes by DOL7. These represented several key pathways in immune development, mainly centered around interferon signaling, the complement cascade, and neutrophil activity. These have previously been shown to play a role in the newborn immune response to infection, but until this study were not identified as central to ontogeny in the first week of life. Importantly, these pathways and nearly 60% of transcriptomic changes were confirmed in a second independent cohort of neonates from Papua New Guinea/Australasia, revealing that neonatal immune development is not random, but follows a precise and possibly purposeful age-specific path.

An unsupervised PPI network was used to integrate the transcriptomic, metabolomics, and proteomic data to reveal a single functional network, highlighting that individual omics datasets are complementary, reporting different facets of the same biological processes. For example, both the transcriptomic and proteomic data confirmed the increase in type I interferon-related functions and the regulation of complement cascades. Importantly, this integration also revealed novel nodes in the PPI network that were not identified by any single-omics dataset on its own, representing novel biological insights, including changes in cellular replication machinery, creatinine metabolism, fibrin clotting cascade, adaptive immunity markers and phagosome activity.

Thus, these systems biology approaches allowed novel insights into the immune developmental trajectory during the first week of life in newborns. Further studies are being conducted to provide insights into the mechanistic differences in the susceptibility of neonates to infection-related disease or death during this critical phase of life. Also, in the context of vaccinology, an integrative systems biology approach is being used to reveal mechanistic insights into the molecular determinants of vaccination efficacy, while taking into account this developmental trajectory.

## Mechanistic Insights Into Host-Pathogen Interactions

Systems biology methods have also been leveraged to study host-pathogen interactions (71). One example is of infection by the obligate human intracellular pathogen *Chlamydia trachomatis*, the major cause of bacterial sexually-transmitted diseases (STDs) and preventable blindness worldwide. This involved a study that coupled transcriptomics and proteomics to assess the macrophage responses to infection with *C. trachomatis* (72). Macrophages were derived from human induced pluripotent stem cells (iPSdMs), which share >95% similarity in terms of gene expression with primary human blood monocyte-derived macrophages, and were able to support the growth of *C. trachomatis* intracellularly to mimic infection *in-vitro*.

Pathway analysis of 2,029 DE genes (from transcriptomics) and 307 DE proteins (from proteomics) at 24 h post-infection, revealed strong interferon α, β, and γ responses, and dysregulation of various Toll-like receptor pathways, the endosomal/vacuolar pathway, energy metabolism, and metabolism of amino acids and nucleotides and inhibition of translation. Most significantly upregulated were genes associated with type I interferon signaling, including key transcription factors such as interferon regulatory factors (IRF)-1, 3, and 7, which are known to contribute to the regulation of type I interferons during *Chlamydia* infection.

Importantly, IRF5 and IL-10RA, not previously characterized for their role in *Chlamydia* infection, were identified as key players in limiting infection in macrophages. Indeed, IRF5^−/−^ and IL-10RA^−/−^ mutant iPSDM cells were both shown to have increased susceptibility to *C. trachomatis* infection. These results, along with numerous other published studies [e.g., (73–77)], demonstrate that multi-omics integration using PPI networks can reveal novel insight into the factors that play a significant role in the host immune response to infections.

## Mechanism-Based Biomarkers for Disease Diagnosis and Prognosis Prediction

Systems biology analyses have also led to insights into mechanisms underlying disease prognosis and prediction of diagnostic biomarkers. One such study of the enteric pathogen *Salmonella enterica sv. Typhimurium* (78) involved the use of transcriptomics to compare gene expression in HIV patients with and without severe invasive non-typhoidal *Salmonella* (iNTS) infections, as well as HIV patients with other acute bacterial infections (including *E. coli* and *Streptococcus pneumoniae*). Initially, 1,200 genes were upregulated in HIV patients with iNTS and with other acute bacterial infections, compared to HIV patients without a bacterial infection. However, genes upregulated in patients with non-*Salmonella* acute infections showed enrichment for pathways typically associated with innate immune/inflammatory responses, while conversely the gene expression response in patients with iNTS could be explained by upregulation of genes that are associated with suppression of inflammation (NFKBIB, PI3K, REL, SIGIRR, SOCS4, SOCS7). This lack of innate immune response and viral signature, which was subsequently shown to be consistent with increased viral load (79), leading to insights into the poor prognosis of HIV patients with iNTS.

These types of analyses were also used to explore immune manipulation using host defense (antimicrobial) peptides. Such peptides selectively modulate the innate immune response and protect against infection, and are produced by many organisms to defend against infections (80). Furthermore, novel small innate defense regulator (IDR) peptides have been shown to be effective in animal models against antibiotic resistant bacteria, tuberculosis, cerebral malaria, pre-term birth and inflammation (81, 82). To better understand the cellular cascade that occurs after these IDR peptides enter the cell, transcriptional changes were assessed in human monocytes and peripheral blood mononuclear cells (83). The biological relevance of these gene expression changes was assessed using pathway over-representation, TFBS analysis, and network analysis with NetworkAnalyst, implicating 11 pathways including the p38, Erk1/2, and JNK mitogen-activated (MAP)-kinases, NFκB, two Src family kinases, and more than 15 transcription factors [including NFκB (most subunits), Creb, IRF4, AP-1, AP-2, Are, E2F1, SP1, Gre, and STAT3]. NetworkAnalyst showed that some of the top connected hub proteins within networks constructed from dysregulated genes were involved in the functioning of MAP kinases and induction of chemokines, anti-inflammatory pathways particularly TGFβ, and type I interferon responses. These highly connected hubs reveal mechanistic insights and could potentially represent diagnostic or treatment biomarkers. Ultimately, a similar approach can be utilized to evaluate any agent perturbing cellular function, including immunomodulators and vaccines, and can define biomarkers differentiating between responders and non-responders.

## Drug Discovery and Repurposing

Systems biology techniques have been applied to aid in drug discovery and repurposing of existing agents for the treatment of cancers, bacterial and viral infections, and genetic disorders (84). One such study aimed at finding better therapeutics for cystic fibrosis (CF) utilized transcriptomics to study immortalized CFTR^−/−^ (cystic fibrosis transmembrane regulator) epithelial cells stimulated for hyperinflammation, a state known to lead to deterioration of lung function in CF patients (85). Genes differentially expressed between CFTR^−/−^ cells and corrected variants were submitted to InnateDB for analysis and integration with PPI networks. This revealed the interconnectivity of the CFTR and innate immune networks through the PRKAA1 (AMP kinase)/AKT1 and HSPB1 pathways. Genes within this network were then submitted to DrugBank (86), allowing for the identification of the diabetes drug Metformin as an AMP kinase activator, which was then tested *in-vitro* and shown to reduce inflammation by ~50%. DE genes between CFTR^−/−^ cells and corrected variants also included 54 genes involved in autophagy. In disease states, autophagy is an adaptive response to stress that favors infection survival and resolution (87). Follow up studies confirmed that CFTR mutant cells demonstrated arrested autophagy. It was then demonstrated that the antimicrobial peptide IDR-1018 resolved this arrested autophagy state and reduced inflammation. These genes also revealed a strong upregulation of ER stress and unfolded protein response pathways, through activation of the IRE-1 pathway (88). Follow up studies showed that salubrinal, an inhibitor of negative regulator GADD34, upregulated this pathway and suppressed inflammation. Thus, through these systems biology-based studies, novel pharmaceuticals (IDR-1018) and 2 existing drugs (Metformin and salubrinal) were identified as potential treatments for CF-related hyperinflammation. As such, along with numerous other studies [e.g., (89–92)], it has been shown that integrating omics datasets using resources such as InnateDB and DrugBank can reveal potential drug targets for improved therapies.

## Discussion and the Future

The analyses outlined in this article merely scratch the surface of what is possible using systems biology and high-throughput omics techniques to study the immune system, e.g., the major tools described here (43, 44, 50–52) have been used and cited more than 1,500 times. The above-described examples highlight that using unbiased multi-omics experiments in conjunction with incisive bioinformatics tools, such as PPI network integration, one can go beyond the hypothesis-testing scientific method to use unbiased omics data to generate fundamentally new hypotheses and develop new biological insights. Ultimately such studies should lead to the development of novel diagnostics, individualized therapies for diseases and vaccines. Furthermore, systems biology approaches can provide invaluable insights to inform the stratification of individuals with the same syndrome but different underlying mechanisms, the diagnosis of disease and/or flare-ups, ongoing development of new vaccines and/or adjuvants as well as immune-based therapeutics providing insights into the optimal strategies for delivery of interventions.

## Author Contributions

BD, MS, and AB all contributed to writing of the first draft of this article. AL performed data analysis of the ontogeny study and provided valuable feedback through the writing process. RH supervised all authors, edited the manuscript and provided critical insights and feedback. All authors contributed to the article and approved the submitted version.

## Conflict of Interest

The authors declare that the research was conducted in the absence of any commercial or financial relationships that could be construed as a potential conflict of interest.

## Abbreviations

CF: cystic fibrosis
CFTR: cystic fibrosis transmembrane regulator
DE: differentially expressed
DOL: day of life
IDR: innate defense regulator
iNTS: invasive non-typhoidal *Salmonella*
MAP: mitogen-activated
PPI: protein-protein interaction
TFBS: transcription factor binding site.
